# Increasing 1,4-Diaminobutane Production in *Escherichia coli* by Optimization of Cofactor PLP and NADPH Synthesis

**DOI:** 10.3390/molecules29133094

**Published:** 2024-06-28

**Authors:** Tong Sun, Yongcan Zhao, Jinjin Wang, Wenke Kang, Xiangxiang Sun, Yanling Sun, Meixue Chu, Zhengyu Liu, Fuping Lu, Ming Li

**Affiliations:** 1 Key Laboratory of Industrial Fermentation Microbiology (Tianjin University of Science and Technology), Ministry of Education, Tianjin 300457, China; aatong9@163.com (T.S.); cc13053767790@163.com (Y.Z.); jinjw1437@163.com (J.W.); k17526620717@163.com (W.K.); sunxx99@outlook.com (X.S.); 13686327051@163.com (Y.S.); chumeixue@163.com (M.C.); liuzhengyu397@163.com (Z.L.); 2 College of Biotechnology, Tianjin University of Science and Technology, Tianjin 300457, China

**Keywords:** PLP, NADPH, 1,4-diaminobutane, cofactor

## Abstract

1,4-diaminobutane is widely used in the industrial production of polymers, pharmaceuticals, agrochemicals and surfactants. Owing to economic and environmental concerns, there has been a growing interest in using microbes to produce 1,4-diaminobutane. However, there is lack of research on the influence of cofactors pyridoxal phosphate (PLP) and NADPH on the synthesis of 1,4-diaminobutane. PLP serves as a cofactor of ornithine decarboxylase in the synthesis of 1,4-diaminobutane. Additionally, the synthesis of 1 mol 1,4-diaminobutane requires 2 mol NADPH, thus necessitating consideration of NADPH balance in the efficient synthesis of 1,4-diaminobutane by *Escherichia coli*. The aim of this study was to enhance the synthesis efficiency of 1,4-diaminobutane through increasing production of PLP and NADPH. By optimizing the expression of the genes associated with synthesis of PLP and NADPH in *E. coli*, cellular PLP and NADPH levels increased, and the yield of 1,4-diaminobutane also increased accordingly. Ultimately, using glucose as the primary carbon source, the yield of 1,4-diaminobutane in the recombinant strain NAP19 reached 272 mg/L·DCW, by increased 79% compared with its chassis strain.

## 1. Introduction

The compound 1,4-diaminobutane, also known as putrescine, is not only involved in cellular metabolism and growth [1,2,3], but also a monomer material used in industry to produce PA46, PA410 and so on [4]. The market demand for 1,4-diaminobutane is growing rapidly due to its high industrial value. At present, 1,4-diaminobutane is mainly produced from petroleum. Industrial scale production of 1,4-diaminbutane mainly relies on chemical synthesis by the addition of hydrogen cyanide to acrylanitrile to obtain succinonitrile, and then hydrogenation of succinonitrile under harsh reaction conditions. The process requires non-renewable petrochemicals as raw materials, as well as expensive catalysts, which can put pressure on environmental protection [5,6]. Therefore, it is necessary to develop a new green production process for 1,4-diaminobutane. By constructing high-yield 1,4-diaminobutane engineered strains, production of 1,4-diaminobutane via microbial fermentation has environmental advantages and represents the future direction of its industrialization.

Engineered strains for the production of 1,4-diaminobutane have been developed and made some progress. At present, the engineered strains were constructed generally by increasing the expression levels of genes in the 1,4-diaminobutane synthesis pathway [7,8,9], knocking out branching metabolic pathways and the degradation pathways of 1,4-diaminobutane [10,11], and screening for high-activity ornithine decarboxylase (ODC) to enhance the synthesis flux of 1,4-diaminobutane [12,13]. In our laboratory, we constructed a chassis strain, *E. coli* PUT11 (DE3), which knocked out 11 genes related to the branching pathway, degradation pathway, and transporter pathway of 1,4-diaminobutane from *E. coli* MG1655 and integrated the T7 RNA polymerase expression cassette (DE3) from *E. coli* BL21(DE3) into the genome. Compared with the wild strain, the 1,4-diaminobutane synthesis was increased by 1888% [14]. However, the yields are insufficient for industrial production requirements. Therefore, other measures are needed to increase the yield of 1,4-diaminobutane.

In the synthetic pathway of 1,4-diaminobutane from glucose, the synthesis of 1 mol 1,4-diaminobutane requires 2 mol NADPH, which are consumed by NADP^-^ -dependent glutamate dehydrogenase (Gdh) and NADP^-^ -dependent N-acetyl-glutaminyl phosphate reductase (ArgC) [15]. Overexpression the genes of the pentose phosphate (PP) pathway are a common method to increase NADPH levels [16,17,18]. In addition to the PP pathway, by overexpression of pyridine nucleotide transhydrogenase gene (*pntAB*) and NAD^+^ kinase gene (*ppnK*) are also a major pathway to increase NADPH concentration in the strain [19,20,21]. Increasing NADPH synthesis in the 1,4-diaminobutane synthesis pathway is crucial.

**Figure 1:**
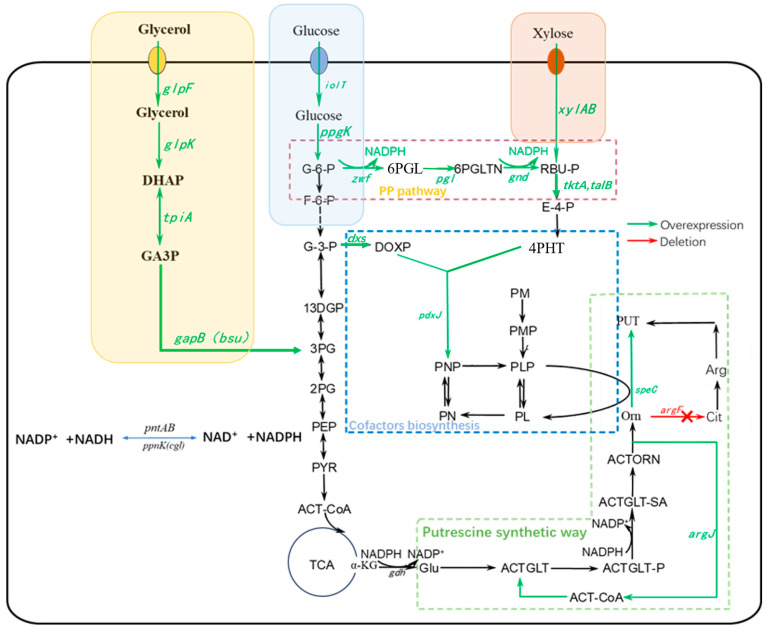
Metabolic pathway design and key metabolic engineering strategies for 1,4-diaminobutane production in *E. coli*. Green arrows indicate overexpressed genes. Red arrows indicate relevant gene deletions. Yellow boxes indicate genes related to glycerol utilization pathway. Blue boxes indicate genes related to glucose utilization pathway and red boxes indicate genes related to D-xylose utilization pathway. *glpF*: glycerol facilitator; *glpK*: glycerol kinase; *tpiA:* triose-phosphate isomerase; *gapB*: glyceraldehyde-3-phosphate dehydrogenase; *iolT*: myoinositol permease; *ppgK*: polyphosphate glucokinase; *xylAB*: xylose isomerase and xylulokinase; *zwf*: ADP(+)-dependent glucose-6-phosphate dehydrogenase; *pgl*: 6-phosphogluconolactonase; *gnd*: 6-phosphogluconate dehydrogenase; *tktA*: transketolase 1; *talB*: transaldolase B; *pdxJ*: pyridoxine 5′-phosphate synthase; *dxs*: 1-deoxy-D-xylulose-5-phosphate synthase; *pntAB*: pyridine nucleotide transhydrogenase subunit alpha and beta; *ppnK*: inorganic polyphosphate/ATP-NAD kinase; *speC*: constitutive ornithine decarboxylase; *argF*: ornithine carbamoyltransferase; *gdh*: Glutamate dehydrogenase; *argC*: N-acetylglutamylphosphate reductase; *argJ*: bifunctional glutamate N-acetyltransferase/amino-acid acetyltransferase; *cgl*: from *Corynebacterium glutamicum*; *bsu*: from *Bacillus subtilis*.

PLP is involved in various enzymatic reactions in *E. coli*, such as decarboxylation, transamination, and racemization in amino acid synthesis [22,23,24]. The de novo pathway of PLP synthesis proceeds from E4P to synthesize 4-phosphohydroxy-l-serine (4PHT), followed by the condensation of 4PHT with 1-deoxy-D-xylulose-5-phosphate (DOXP) catalyzed by pyridoxine 5′-phosphate synthase (PdxJ) to form pyridoxamine 5′-phosphate (PNP), which is further oxidized to PLP [25,26] (Figure 1). Many studies have shown that optimizing the synthesis of PLP can effectively increase the yield of the cadaverine [27,28,29]. PLP is used as a cofactor for ODC, a key enzyme in the synthesis of the 1,4-diaminobutane [30,31]. A moderate increase in PLP supply is important for 1,4-diaminobutane synthesis.

Currently, the construction processes of recombinant strains for 1,4-diaminobutane biosynthesis lack the researches on PLP and NADPH. In this study, overexpression of the genes related to NADPH synthesis, including *pntAB*, *ppnK* and the genes in PP pathway, was used to increase NADPH concentration in the strains to study the impact of NADPH on 1,4-diaminobutane synthesis (Figure 1). The relationship between PLP synthesis and 1,4-diaminobutane production was investigated by increasing PLP synthesis in *E. coli* through overexpression of the genes *pdxJ* and *dxs*, which are involved in the PLP synthesis pathway, and *tktA* and *talB*, which are the genes of the E4P synthesis pathway, a precursor of PLP (Figure 1). Furthermore, the impact of increasing NADPH and PLP synthesis in the chassis strain *E. coli* PUT11 (DE3) on 1,4-diaminobutane production was explored.

## 2. Results

### 2.1. Optimisation of NADPH Synthesis in the 1,4-Diaminobutane Synthetic Pathway

In the 1,4-diaminobutane synthesis pathway, 2 mol NADPH is required to synthesize 1,4-diaminobutane. PntAB and PpnK catalyze NADP^+^ and NADH to generate NADPH. In addition, overexpression of PP pathway could effectively increase NADPH concentration in strains. Therefore, in this study, the key genes involved in NADPH synthesis, *pntAB* and *ppnK*, as well as the genes in the PP pathway, were overexpressed to increase the production of NADPH and thus increased the production of 1,4-diaminobutane (Figure 1).

#### 2.1.1. Overexpression of *ppnK* and *pntAB* Increased NADPH Supply

**Figure 2:**
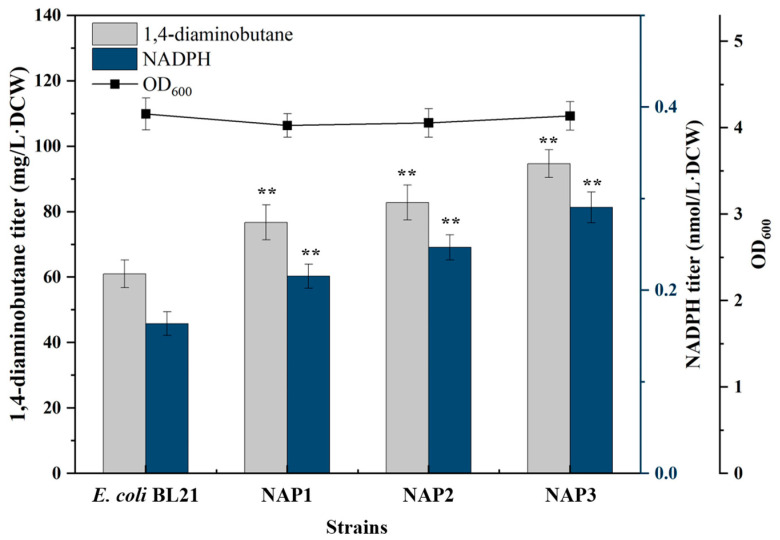
Determination of NADPH and 1,4-diaminobutane titer of NAP1, NAP2 and NAP3. Strain genotypes: NAP1: pETM6-ppnK; NAP2: pETM6-pntAB; NAP3: pETM6-ppnK-pntAB. Data were expressed as means, and error bars indicate standard deviation (*n* = 3 independent experiments. The ** symbol represented *p* < 0.01 compared with their controls).

The cloned *ppnK* and *pntAB* genes were ligated into pETM6 plasmid and transformed into *E. coli* BL21(DE3) to form recombinant strains NAP1 and NAP2. Recombinant strains NAP1 and NAP2 could catalyze NADP^+^ to NADPH by expressing *pntAB* and *ppnK*. NADPH synthesis and 1,4-diaminobutane yield were shown in Figure 2. The NADPH yield of NAP1 and NAP2 was significantly higher than that of the control strain *E. coli* BL21(DE3), and similarly, the yield of 1,4-diaminobutane was also increased companied with the elevation of NADPH, showing that improvement of NADPH can enhance the synthesis of 1,4-diaminobutane. The titer of 1,4-diaminobutane in NAP1 and NAP2 was 77 mg/L·DCW and 83 mg/L·DCW, respectively, which was about 27% and 37% higher than that in the control strain *E. coli* BL21(DE3). In order to furtherly increase the synthesis of NADPH, co-expression plasmid of *pntAB* and *ppnK* was transformed into *E. coli* BL21(DE3) to generate recombinant strain NAP3. The NADPH yield of NAP3 strain was higher, and the 1,4-diaminobutane titer was 95 mg/L·DCW, which was 57% greater than the control strain *E. coli* BL21(DE3) (Figure 2), indicating that the synthesis of 1,4-diaminobutane was positively correlated with NADPH.

#### 2.1.2. Overexpression of PP Pathway Genes Increased NADPH Supply

**Figure 3:**
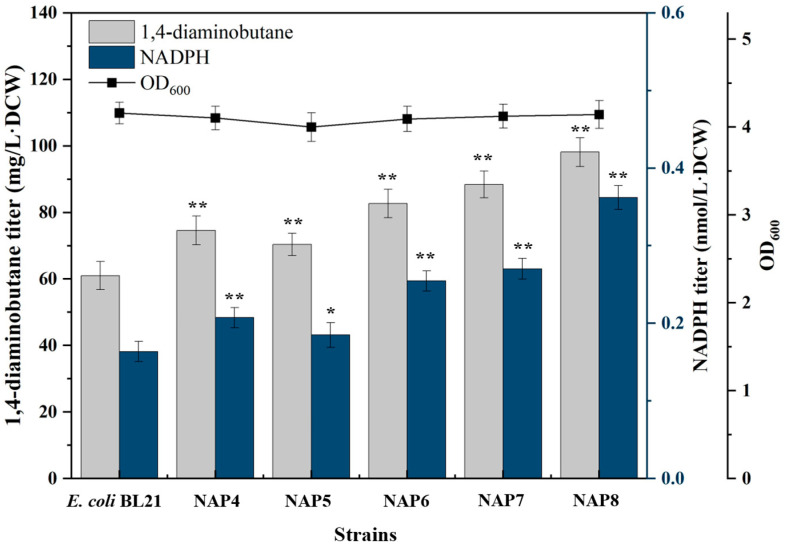
Determination of NADPH and 1,4-diaminobutane titer in NAP4, NAP5, NAP6, NAP7 and NAP8. Strain genotypes: NAP4: pETM6-zwf; NAP5: pETM6-pgl; NAP6: pETM6-gnd; NAP7: pETM6-zwf-pgl; NAP8: pETM6-zwf-gnd-pgl. Data were expressed as means, and error bars indicated standard deviation (*n* = 3 independent experiments. The * symbol represented 0.01 < *p* < 0.05, and ** represented *p* < 0.01 compared with their controls).

In the PP pathway, NADP^+^-dependent glucose-6-phosphate dehydrogenase (Zwf) and NADP^+^-dependent glucose-6-phosphate dehydrogenase (Gnd) can reduce NADP^+^ to NADPH [32,33] (Figure 1). The *zwf,* 6-phosphogluconolactonase (*pgl*) and *gnd* genes of this pathway were cloned into the pETM6 plasmid, and transformed into *E. coli* BL21(DE3) to construct recombinant strains NAP4, NAP5 and NAP6. Recombinant strains NAP4, NAP5 and NAP6 increased NADPH synthesis by expressing *zwf*, *pgl* and *gnd* genes of PP pathway. The result was shown in Figure 3, overexpression of the *zwf*, *pgl* and *gnd* genes could all promote NADPH synthesis, thereby increased the yield of 1,4-diaminobutane. The 1,4-diaminobutane titer of NAP4, NAP5 and NAP6 was 75 mg/L·DCW, 70 mg/L·DCW and 83 mg/L·DCW, respectively, which was 24%, 17% and 38% higher than those of the control strain *E. coli* BL21(DE3). Subsequently, the expression plasmids harbouring *zwf-gnd* and *zwf-gnd-pgl* were transformed into *E. coli* BL21(DE3) to construct recombinant strains NAP7 and NAP8. As shown in Figure 3, with the further improvement of NADPH yield, the synthesis levels of 1,4-diaminobutane were furtherly increased. The titer of 1,4-diaminobutane of NAP7 and NAP8 was 88 mg/L·DCW and 98 mg/L·DCW, respectively, which were 47% and 63% higher than the control strain *E. coli* BL21(DE3). It was proved that overexpression of the genes in the PP pathway could effectively improve NADPH level and 1,4-diaminobutane synthesis.

### 2.2. Optimization of PLP Supply in the 1,4-Diaminobutane Synthesis Pathway

#### 2.2.1. Effect of Exogenous Supplementation of PLP on the Synthesis of 1,4-Diaminobutane in *E. coli*

**Figure 4:**
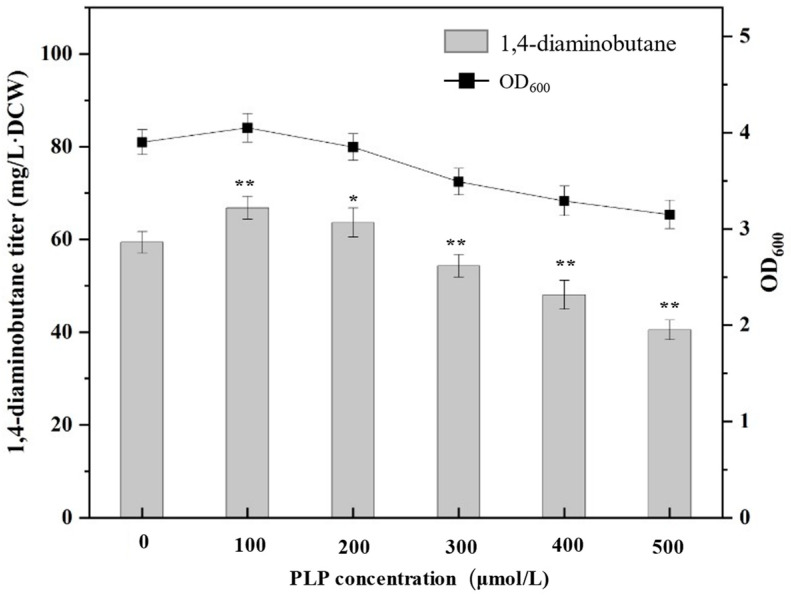
Production of 1,4-diaminobutane and OD_600_ after 24 h fermentation culture with different concentrations of PLP. The concentration of PLP was 100, 200, 300, 400 and 500 μmol/L, and the fermentation supernatant was used to measure the concentration of 1,4-diaminobutane. Data were expressed as means, and error bars indicated standard deviation (*n* = 3 independent experiments. The * symbol represented 0.01 < *p* < 0.05, and ** represented *p* < 0.01 compared with their controls).

Excessive accumulation of PLP in cells can cause certain toxicity and affect cell growth. Therefore, it is first necessary to determine the effect of PLP concentration on the synthesis of 1,4-diaminobutane. PLP of 100, 200, 300, 400 and 500 μmol/L were added to the medium, and the yield of 1,4-diaminobutane in the fermentation supernatant and OD_600_ was measured after 24 h of fermentation culture, as shown in Figure 4. When PLP is not added, the titer of 1,4-diaminobutane of *E. coli* BL21(DE3) is 59 mg/L·DCW, while when PLP is added exogenously at 100 μmol/L, the titer of 1,4-diaminobutane of *E. coli* BL21(DE3) and OD_600_ were the highest, respectively reaching 66 mg/L·DCW and 4. However, with the increase of PLP concentration, both the production of 1,4-diaminobutane and OD_600_ decreased. These results indicated that a reasonable increase of PLP synthesis is beneficial to the synthesis of 1,4-diaminobutane, but not too much, otherwise it will affect the growth of strains and the synthesis of 1,4-diaminobutane.

#### 2.2.2. Enhancing the Synthesis of PLP

**Figure 5:**
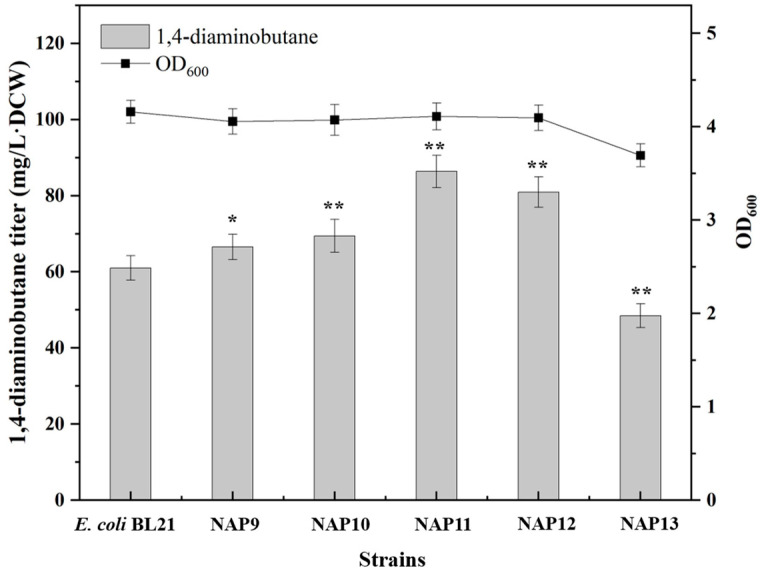
Determination of 1,4-diaminobutane titer in NAP9, NAP10, NAP11, NAP12 and NAP13. Strain genotypes: NAP9: pETM6-pdxJ; NAP10: pETM6-dxs; NAP11: pETM6-tktA; NAP12: pETM6-talB; NAP13: pETM6-pdxJ-dxs-tktA-talB. Data were expressed as means, and error bars indicated standard deviation (*n* = 3 independent experiments. The * symbol represented 0.01 < *p* < 0.05, and ** represented *p* < 0.01 compared with their controls).

The genes *pdxJ* and *dxs*, involved in the PLP synthesis pathway, and the genes *tktA* and *talB*, responsible for the synthesis of PLP precursors E4P, were cloned into the pETM6 plasmid and transformed into *E. coli* BL21(DE3) to generate recombinant strains NAP9, NAP10, NAP11 and NAP12. These recombinant strains all could increase PLP synthesis due to expressing *pdxJ*, *dxs*, *tktA* and *talB* genes related to synthesis of PLP (Figure 1). The results are shown in Figure 5. Overexpression of these genes could increase the synthesis of 1,4-diaminobutane. Overexpression of *pdxJ* and *dxs* genes in the PLP synthesis pathway resulted in 1,4-diaminobutane accumulation levels of 67 mg/L·DCW and 69 mg/L·DCW, respectively, with an increase of 11% and 15% compared with the control strain *E. coli* BL21(DE3). Overexpression of the genes *tktA* and *talB* resulted in 1,4-diaminobutane accumulation levels of 86 mg/L·DCW and 80 mg/L·DCW, with an increase of 44% and 35%, respectively, compared with the control strain *E. coli* BL21(DE3). This showed that increasing the PLP significantly increased 1,4-diaminobutane production.

So as to furtherly enhance PLP synthesis, *pdxJ, dxs*, *tktA* and *talB* were cloned into pETM6 to construct the the PLP synthesis module plasmid pETM6-pdxJ-dxs-tktA-talB. This plasmid was transformed into *E. coli* BL21(DE3) to create the recombinant strain NAP13. The results showed that when enhancing the expression of PLP synthesis module, the accumulation of 1,4-diaminobutane not only did not increase, on the contrary, the yield of 1,4-diaminobutane decreased by approximately 22% compared with the control strain *E. coli* BL21(DE3) (Figure 5). At the same time, OD_600_ also decreased. It was possible that after enhancing the expression of PLP synthesis module, PLP concentration was too high, which led to toxicity to the cells and a reduction in 1,4-diaminobutane production. This was in line with the previous findings that an excessively high PLP concentration did not improve 1,4-diaminobutane production, inversely, it reduced its accumulation.

### 2.3. Overexpression of 1,4-Diaminobutane Synthesis Module

The bifunctional N-acetyltransferase enzyme encoded by *argJ* in *C. glutamicum* catalyzes the conversion of N-acetylornithine to ornithine through deacetylation, and simultaneously transfers the acetyl group produced from this reaction to glutamic acid to form N-acetylglutamic acid, thus facilitating the recycling of the acetyl group [34,35,36]. Therefore, the key gene *speC* gene encoding ODC and *argJ* were cloned into the pRSM3 plasmid to construct a 1,4-diaminobutane synthesis module plasmid pRSM3-speC-argJ.

**Figure 6:**
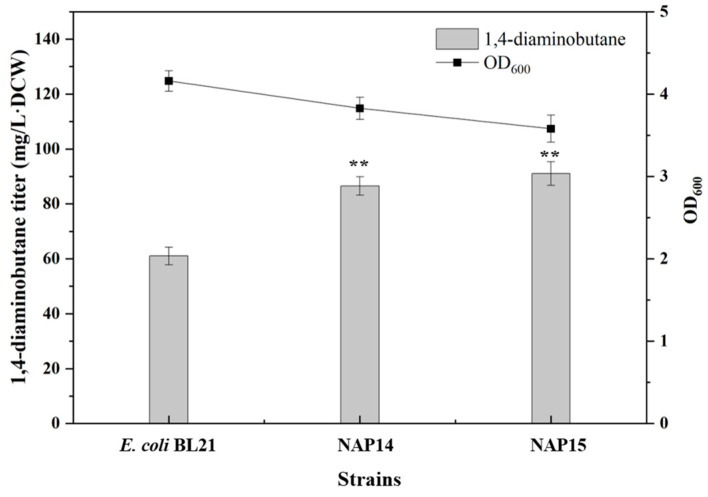
Determination of 1,4-diaminobutane titer in NAP14 and NAP15. Strain genotypes: NAP14: pETM6-pdxJ-dxs-tktA-talB and pRSM3-speC-argJ; NAP15: NAP14 harbouring the pCDM4-pntAB-ppnK and pACM4-zwf-gnd-pgl. Data were expressed as means, and error bars indicated standard deviation (*n* = 3 independent experiments. The ** represented *p* < 0.01 compared with their controls).

The strain NAP14 was constructed by co-transforming the 1,4-diaminobutane synthesis module plasmid pRSM3-speC-argJ and the PLP synthesis module plasmid pETM6-pdxJ-dxs-tktA-talB into *E. coli* BL21(DE3), and the yield of 1,4-diaminobutane was detected. The results showed that NAP14 produced 86 mg/L·DCW of 1,4-diaminobutane, with a 44% increase compared to the control strain *E. coli* BL21(DE3) (Figure 6). As ODC is a crucial gene in the synthesis of 1,4-diaminobutane, increasing the expression of ODC in the strain allows for the extensive utilization of PLP and decreases PLP’s toxicity.

Afterward, the NADPH synthesis module plasmid pCDM4-pntAB-ppnK and pACM4-zwf-gnd-pgl were constructed and transferred into NAP14 to obtain strain NAP15. The results are shown in Figure 6, the 1,4-diaminobutane titer of NAP15 was 91 mg/L·DCW, with an increase of 51% compared with the control strain *E. coli* BL21(DE3), which proved that with increasing synthesis of PLP and NAPDH, overexpression of the 1,4-diaminobutane synthesis module could lead to an increase in 1,4-diaminobutane production. At the same time, the density of NAP15 strain decreased, speculating that the four plasmids in the same strain increased the metabolic burden of the strain and inhibited the growth of the strains.

### 2.4. Effect of Increasing NADPH and PLP Synthesis on 1,4-Diaminobutane Yield When Utilizing Different Carbon Sources

#### Overexpression of Carbon Source Utilization Pathway Genes

**Figure 7:**
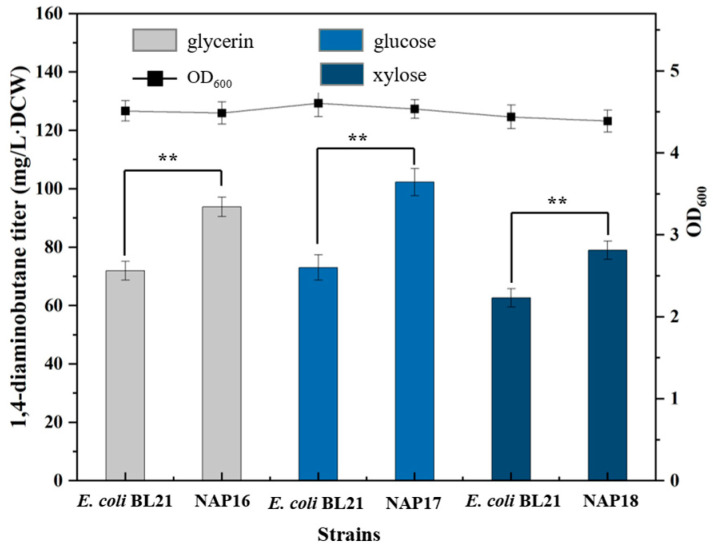
Determination of 1,4-diaminobutane titer in NAP16, NAP17 and NAP18. Strain genotypes: NAP16: pETM6-glpFK-tpiA-gapB; NAP17: pETM6-iolT-ppgK; NAP18: pETM6-xylAB. Grey represented adding 1 g/L of glycerol to the medium; Light blue represented adding 1 g/L of glucose to the medium; Dark blue represented adding 1 g/L of xylose to the medium. Data were expressed as means and error bars indicated standard deviation (*n* = 3 independent experiments. The ** represented *p* < 0.01 compared with their controls).

Carbon source is one of the factors that must be considered in fermentation production [37,38,39]. In this study, glycerol utilization pathway, glucose utilization pathway, and xylose utilization pathway plasmids were constructed, respectively, and transformed into *E. coli* BL21(DE3) to construct recombinant strains NAP16, NAP17 and NAP18. The strains were cultured in SOB medium containing 1 g/L glycerol, 1 g/L glucose and 1 g/L xylose, respectively. As shown in Figure 7, the 1,4-diaminobutane titer of NAP16, NAP17 and NAP18 were 93 mg/L·DCW, 102 mg/L·DCW and 78 mg/L·DCW with an increase of 30%, 40% and 24% compared with the control strain *E. coli* BL21(DE3). These results showed that increasing utilization of the carbon source by overexpressing their genes could significantly increase the yield of the target products, and the recombinant strains overexpressing the glucose pathway genes had the most obvious effect.

### 2.5. Fermentation Results of Recombinant Strains

**Figure 8:**
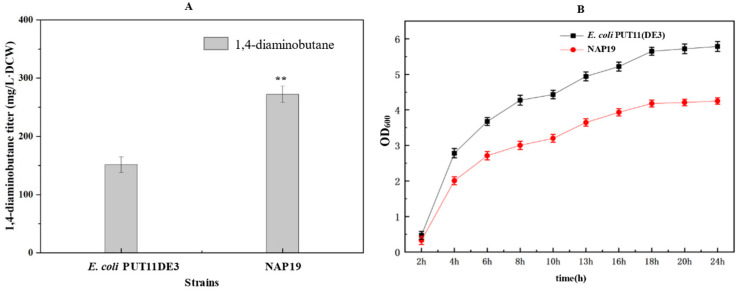
Determination of 1,4-diaminobutane titer in NAP19. (**A**) 1,4-diaminobutane titer in NAP19; (**B**) Cell growth of the recombinant NAP19 strain and the control strain. Strain genotypes NAP19: *E. coli* PUT11(DE3) harbouring the pRSM3-iolT-ppgk, pTrc99A-speC-argJ, pACM4-zwf-gnd-pgl, pCDM4-pdxJ-dxs-tktA-talB-pntAB-ppnk. Data were expressed as means and error bars indicated standard deviation (*n* = 3 independent experiments. The ** represented *p* < 0.01 compared with their controls).

The constructed glucose utilization pathway plasmid, NADPH and PLP synthesis plasmid, and ornithine decarboxylase plasmid were co-transformed into *E. coli* PUT11(DE3) to construct the strain NAP19. *E. coli* PUT11(DE3) has been knocked out 11 genes which were associated with the pathways for 1,4-diaminobutane degradation and transport and knocked in DE3 sequence from *E. coli* BL21(DE3) in PuuR operon site. The fermentation results are shown in Figure 8. NAP19 finally achieved a 1,4-diaminobutane titer of 272 mg/L·DCW, there was a 79% increase compared to the control strain *E. coli* PUT11(DE3). The results showed that in the strain overexpressing the genes of glucose pathway, enhancing the PLP and NADPH synthesis could increase the yield of 1,4-diaminobutane. However, NAP19 exhibited slower growth compared with the control strain, *E. coli* PUT11(DE3), which was attributed to the metabolic burden imposed by the presence of four free plasmids containing multi-gene expression cassettes, ultimately impacting the strain’s growth efficiency.

## 3. Discussion

NADPH and PLP are the crucial cofactors in the biosynthetic pathway of 1,4-diaminobutane. The construction process of the reported high-yield 1,4-diaminobutane recombinant strains lacked researches on the synthesis of NADPH and PLP. Furthermore, in this study, the effect on the yield of 1,4-diaminobutane was determined by optimizing the PLP and NADPH synthesis pathways in *E. coli*.

Synthesizing 1 mol 1,4-diaminobutane in the pathway requires the consumption of 2 mol NADPH. Overexpressing key genes involved in NADPH synthesis, such as *pntAB* and *ppnK*, along with genes in the PP pathway, could enhance NADPH production and increased the yield of 1,4-diaminobutane. Ultimately, the accumulated amounts in the recombinant strains NAP3 and NAP8 were 95 mg/L·DCW and 98 mg/L·DCW, respectively, showing increases of 57% and 63% compared to the control strains *E. coli* BL21(DE3). This demonstrated that boosting NADPH synthesis in *E. coli* could effectively enhance the production of 1,4-diaminobutane.

PLP is the cofactor of ODC. Within a certain range of PLP concentrations, the synthesis of 1,4-diaminobutane is positively correlated with the PLP content in the cells. When the PLP content exceeds a certain range, cell growth is inhibited, leading to a decrease in 1,4-diaminobutane synthesis. Hence, optimizing the PLP content is essential when constructing high-yield 1,4-diaminobutane strains. Overexpressing genes involved in PLP synthesis, such as *pdxJ* and *dxs*, and genes involved in the synthesis of PLP precursors E4P, like *tktA* and *talB*, could increase the synthesis of 1,4-diaminobutane. However, when *pdxJ, dxs, tktA* and *talB* were co-expressed in the recombinant strain NAP13, the accumulation of 1,4-diaminobutane inversely decreased. At the same time, OD_600_ also decreased because enhancing the expression of PLP synthesis led to toxicity to the cells. This showed that PLP could promote the biosynthesis of 1,4-diaminobutane in a certain concentration range, otherwise, the biosynthesis of 1,4-diaminobutane was inhibited.

The fermentation of *E. coli* using glucose, glycerol and xylose was optimized, and the fermentation results showed that the 1,4-diaminobutane produced by the strain using glucose was the highest. Therefore, NADPH and PLP synthesis pathway plasmids, glucose utilization plasmids, and 1,4-diaminobutane synthesis plasmids were transferred to *E. coli* PUT11(DE3), which knocked out 11 genes associated with 1,4-diaminobutane degradation and transport pathways and incorporated the T7 RNA polymerase of *E. coli* BL21(DE3). Finally, the yield of the recombinant strain NAP19 reached 272 mg/L·DCW, which was 79% higher than that of the control strain *E. coli* PUT11(DE3). This suggested that the production of 1,4-diaminobutane could be increased by enhancing the synthesis of NADPH and PLP in the engineering strain. The effect of NADPH and PLP concentrations on 1,4-diaminobutane yield should also be considered.

This study revealed the importance of NADPH and PLP synthesis for the production of 1,4-diaminobutane. Although the yield of 1,4-diaminobutane in recombinant strains was not high, these results proved that the synthesis of NADPH and PLP in recombinant strains could effectively improve the yield of 1,4-diaminobutane, which provided a theoretical basis for the construction of high-yielding 1,4-diaminobutane recombinant strains.

## 4. Materials and Methods

### 4.1. Strain, Plasmid and Culture Conditions

*E. coli* str. K12 substr. MG1655 was used as the starting strain for genome manipulation. *E. coli* BL21(DE3) and *E. coli* PUT11(DE3) were used for the synthesis of 1,4-diaminobutane. The constructed strains and genetic phenotypes are shown in Table 1.

During plasmid and strain construction, cells were grown on LB solid plates containing appropriate antibiotics. When needed, antibiotics were added at the following concentrations: 50 mg/mL kanamycin (Kan), 100 mg/mL ampicillin (Amp), 25 mg/mL chloramphenicol (Cl) and 50 mg/mL streptomycin (Str).

The target genes in this study were amplified using matched primers using relevant host genomic DNA as a template. Heterologous genes were amplified using primers from *E. coli* MG1655, *C. glutamicum* 13032 and *B. subtilis* genomes as templates, respectively. All of the primers used in this study were listed in Supplementary Table S1.

### 4.2. Plasmids Construction

The pETM6 plasmid and interest genes were digested using *Spe*I and *Nde*I and subsequently ligated with a ligase to construct the pETM6 expression plasmid.

The pACM4, pCDM4, pRSM3 and pETM6 plasmids have three major cleavage sites: *Bln*Ⅰ, *Nhe*I and *Sal*I, and after cleavage they will produce the same CTAG sticky end. Firstly, the interest gene was cloned into the *Nde*I and *Spe*I of pETM6. Then, interest gene expression cassette was digested using *Bln*Ⅰ and *Sal*I from a constructed plasmid and ligated into between *Nhe*I and *Sal*I of the other plasmid by isocaudamers for achieving a tandem linkage of genes (Supplementary Figure S1). The same method was used to tandem genes on pACM4, pCDM4 and pRSM3. The constructed plasmids are shown in Supplementary Table S2.

### 4.3. Shake Flask Culture and Fermentation

The constructed engineering strains were inoculated from solid medium into LB liquid medium containing corresponding antibiotics, and incubated at 37 °C, 220 r/min for 14–16 h as seed culture. The seed was inoculated into 500 mL shake flasks containing fermentation medium according to 1% inoculum volume, and the corresponding antibiotics was added.

Fermentation medium:

(1) SOB medium (Super Optimal Broth): 20 g/L tryptone, 5 g/L yeast extract, 0.5 g/L NaCl, add 10 mL of 250 mmol/L KCl solution, and adjust pH to 7.0. Supplemented with 1 g/L carbon source. The solution was autoclaved at 115 °C for 20 min. 5 mL of sterilised 2 mol/L MgCl_2_ was added before use.
(2) R/2 medium: 5 g/L yeast extract, 1 g/L peptone, 2 g/L (NH_4_)_2_HPO_4_, 6.75 g/L KH_2_PO_4_, 0.85 g/L citric acid, 0.7 g/L MgSO_4_·7H_2_O, 10 g/L glucose, 3 g/L (NH_4_)_2_SO_4_ and 5 mL/L trace metal solution. The pH was adjusted to 6.8 and autoclaved at 115 °C for 20 min. The trace metal solution contained 5 mol/L HCl, 10 g/L FeSO_4_·7H_2_O. 2.25 g/L ZnSO_4_·7H_2_0, 1 g/L CuSO_4_·5H_2_O, 0.5 g/L MnSO_4_·5H_2_O, 1.23 g/L Na_2_B_4_O_7_·10H_2_O, 2 g/LCaCl_2_·2H_2_O and 0.1 g/L (NH_4_)_6_Mo_7_O_24_.

### 4.4. Analytical Method

Strain growth was monitored by measuring absorbance at 600 nm (OD_600_) using a UV spectrophotometer. The dry cell weights (DCW) were determined by heating the samples to a constant weight at 80 °C.

NADPH concentrations were determined based on the glucose dehydrogenase cycle reaction using the CheKine^TM^ Micro Coenzyme II NADP(H) Assay Kit (Abbkine Scientific, CHN. https://www.abbkine.com/product/chekine-micro-coenzyme-%e2%85%a1-nadph-assay-kit-ktb1010/, accessed on 5 June 2024). The strains samples were harvested by centrifugaed and washed with PBS. The pellet cells were added 100 µL of NADPH Extraction Buffer and broken by ultrasonic crushing (power 200 W, ultrasonic 3 s, interval 10 s, repeated 30 times). And then, the extracts were heated at 60 °C for 5 min, 20 µL of Assay Buffer was added, followed by the addition of an equal volume of reverse extraction solution to neutralize it. Vortexed briefly, the samples were centrifuged at 14,000 r/min for 5 min at 4 °C. The supernatant was transferred to a new microcentrifuge tube, and the NADPH content was measured at 450 nm.

1,4-diaminobutane was derived with dansyl chloride so that it can be detected by ultraviolet at 254 nm [40]. After 1 mL sample was centrifuged, 500 μL supernatant was taken into a microcentrifuge tube. Added 500 μL saturated NaHCO_3_, and adjusted the pH to 10 with saturated NaOH. Added 1 mL dansyl chloride (5 g/L) for derivative reaction. Incubated the tubes at 60 °C for 30 min, and then 2 mL anhydrous ether was added for extraction. Took the top organic phase into the microcentrifuge tube, dried, and added of 500 μL acetonitrile to dissolve. The concentration of 1,4-diaminobutane was detected by HPLC. The chromatographic column was Agilent porpshell EC-C18 column (4.6 mm × 250 mm, particle size 4 μm). The detection method was as follows: 30 °C, UV wavelength 254 nm, sample size 10 uL, mobile phase A as ultra-pure water, mobile phase B as acetonitrile, flow rate 0.7 mL/min, gradient elution: 0–5 min: 55% to 70% B; 5–10 min: 70% B; 10–15 min: 70 to 95% B; 15–20 min: 95% B; 20-25 min: 95 to 55% B. The retention time of 1,4-diaminobutane is about 14 min. Chromatographic peak of 1,4-diaminobutane is shown in Supplementary Materials Figure S2.

### 4.5. Statistical Analysis

The data in this study represented the mean values and standard deviations of three independent experiments. The significant differences in data were determined via a one-way analysis of variance. The value of 0.01 < *p* < 0.05 indicated a significant difference compared with their controls, and a *p* value < 0.01 indicated a highly significant difference compared with their controls.

## 5. Conclusions

This study optimized the supply of NADPH and PLP in the 1,4-diaminobutane biosynthesis pathway of *E. coli* and confirmed the positive effects of increased NADPH and PLP synthesis on 1,4-diaminobutane production. Ultimately transferring NADPH and PLP synthetic plasmids and glucose utilization plasmids into the *E. coli* PUT11(DE3) strain, the resulting strain NAP19 accumulated 272 mg/L·DCW of 1,4-diaminobutane. Increasing the supply of the cofactors PLP and NADPH was shown to be of significant importance for the synthesis of 1,4-diaminobutane. It laid a foundation for the construction of high-yielding 1,4-diaminobutane recombinant strains.

## Figures and Tables

**Table 1:** List of strains.

Strains	Genotypes	Source
*E. coli str*. K12 substr. MG1655	Template for gene from *E.coli* cloning	Kept in our lab
*C. glutamicum* 13032	Wild type, donor of *ppnk*, *argJ* gene	Kept in our lab
*B. subtilis subsp. subtilis str. 168*	Wild type, donor of *gapB* gene	Kept in our lab
*E. coli* PUT11(DE3)	K12 MG1655△*argR*△*patA*△*puuA*△*speED*△*speG*△*puuP*△*argF*△*ydcSTUV*△*potFGHI*△*plaP::*DE3	Kept in our lab
NAP1	K12 harbouring pETM6-ppnk, Amp	This study
NAP2	K12 harbouring pETM6-pntAB, Amp	This study
NAP3	K12 harbouring pETM6-ppnk -pntAB, Amp	This study
NAP4	BL21(DE3) harbouring pETM6-zwf, Amp	This study
NAP5	BL21(DE3) harbouring pETM6-pgl, Amp	This study
NAP6	BL21(DE3) harbouring pETM6-gnd, Amp	This study
NAP7	BL21(DE3) harbouring pETM6-zwf-pgl, Amp	This study
NAP8	BL21(DE3) harbouring pETM6-zwf-pgl-gnd, Amp	This study
NAP9	BL21(DE3) harbouring pETM6-pdxJ, Amp	This study
NAP10	BL21(DE3) harbouring pETM6-dxs, Amp	This study
NAP11	BL21(DE3) harbouring pETM6-tktA, Amp	This study
NAP12	BL21(DE3) harbouring pETM6-talB, Amp	This study
NAP13	BL21(DE3) harbouring pETM6-pdxJ-dxs-tktA-talB, Amp	This study
NAP14	NAP13 harbouring pRSM3-speC-argJ, Kan	This study
NAP15	NAP14 harbouring pCDM4-pntAB-ppnK and pACM4-zwf-gnd-pgl, Cl	This study
NAP16	BL21(DE3) harbouring pETM6-glpFK-tpiA-gapB, Amp	This study
NAP17	BL21(DE3) harbouring pETM6-iolT-ppgK, Amp	This study
NAP18	BL21(DE3) harbouring pETM6-xylAB, Amp	This study
NAP19	PUT11(DE3) harbouring the pRSM3-glpFK-tpiA-gapB, pTrc99A-speC-argJ, pACM4-zwf-gnd-pgl, pCDM4-pdxJ-dxs-tktA-talB-pntAB-ppnk. Kan, Amp, Cl, Str	This study

## Data Availability

The data are available from the corresponding author upon request.

## Supplementary Materials

The following supporting information can be downloaded at: https://www.mdpi.com/article/10.3390/molecules29133094/s1, Table S1: Primers used in this study. Table S2: List of plasmids. Figure S1: Flow chart of tandem carrier constructed by isocaudamers. Figure S2: Determination of 1,4-diaminobutane by HPLC. (A) the chromatographic peak of 1,4-diamino-butane standard, which peaked in about 14 min. (B) the chromatographic peak of the sample, which peaked in about 14 min.

## References

[1] Zhang X., Zhao P., Kuai J., Chang C., Yuan Q. (2024). Spermidine alleviates lipopolysaccharide-induced myocardial injury in mice by suppressing apoptosis, ROS production and ferroptosis. J. South. Med. Univ..

[2] Holbert C.E., Cullen M.T., Casero R.A., Stewart T.M. (2022). Polyamines in cancer: Integrating organismal metabolism and antitumour immunity. Nat. Rev. Cancer.

[3] Pérez-Pertejo Y., García-Estrada C., Martínez-Valladares M., Murugesan S., Reguera R.M., Balaña-Fouce R. (2024). Polyamine Metabolism for Drug Intervention in Trypanosomatids. Pathogens.

[4] Lee P.C., Kim S.Y., Ko Y.K., Ha J.U., Jeoung S.K., Shin D., Kim J.H., Kim M.G. (2022). Tribological Properties of Polyamide 46/Graphene Nanocomposites. Polymers.

[5] Li Z., Liu J.Z. (2017). Transcriptomic Changes in Response to Putrescine Production in Metabolically Engineered *Corynebacterium glutamicum*. Front. Microbiol..

[6] Sanders J., Scott E., Weusthuis R., Mooibroek H. (2007). Biorefinery as the bioinspired process to bulk chemicals. Macromol. Biosci..

[7] Son J., Sohn Y.J., Baritugo K.A. (2023). Recent advances in microbial production of diamines, aminocarboxylic acids, and diacids as potential platform chemicals and bio-based polyamides monomer. Biotechnol. Adv..

[8] Yang S.-C., Ting W.-W., Ng I.-S. (2022). Effective whole cell biotransformation of arginine to a four-carbon diamine putrescine using engineered *Escherichia coli*. Biochem. Eng. J..

[9] Qian Z.G., Xia X.X., Lee S.Y. (2009). Metabolic engineering of *Escherichia coli* for the production of putrescine: A four carbon diamine. Biotechnol. Bioeng..

[10] Li Z., Shen Y.P., Jiang X.L., Feng L.S., Liu J.Z. (2018). Metabolic evolution and a comparative omics analysis of *Corynebacterium glutamicum* for putrescine production. J. Ind. Microbiol. Biotechnol..

[11] Nguyen A.Q., Schneider J., Wendisch V.F. (2015). Elimination of polyamine N-acetylation and regulatory engineering improved putrescine production by *Corynebacterium glutamicum*. J. Biotechnol..

[12] Hong E.Y., Kim J.Y., Upadhyay R., Park B.J., Lee J.M., Kim B.G. (2018). Rational engineering of ornithine decarboxylase with greater selectivity for ornithine over lysine through protein network analysis. J. Biotechnol..

[13] Tan Q., Gou L., Fan T.P., Cai Y. (2024). Enzymatic properties of ornithine decarboxylase from Clostridium aceticum DSM1496. Biotechnol. Appl. Biochem..

[14] Li M., Lu F., Sun X. (2024). Chassis Strain of Escherichia coli Producing 1,4-Diaminobutane and Its Application.

[15] Schneider J., Wendisch V.F. (2011). Biotechnological production of polyamines by bacteria: Recent achievements and future perspectives. Appl. Microbiol. Biotechnol..

[16] Noh M., Yoo S.M., Kim W.J., Lee S.Y. (2017). Gene Expression Knockdown by Modulating Synthetic Small RNA Expression in *Escherichia coli*. Cell Syst..

[17] Jensen J.V., Eberhardt D., Wendisch V.F. (2015). Modular pathway engineering of *Corynebacterium glutamicum* for production of the glutamate-derived compounds ornithine, proline, putrescine, citrulline, and arginine. J. Biotechnol..

[18] Kim S.Y., Lee J., Lee S.Y. (2015). Metabolic engineering of *Corynebacterium glutamicum* for the production of L-ornithine. Biotechnol. Bioeng..

[19] Sauer U., Canonaco F., Heri S., Perrenoud A., Fischer E. (2004). The soluble and membrane-bound transhydrogenases UdhA and PntAB have divergent functions in NADPH metabolism of *Escherichia coli*. J. Biol. Chem..

[20] Kawai S., Mori S., Mukai T., Suzuki S., Yamada T., Hashimoto W., Murata K. (2000). Inorganic Polyphosphate/ATP-NAD kinase of Micrococcus flavus and Mycobacterium tuberculosis H37Rv. Biochem. Biophys. Res. Commun..

[21] Shi F., Li Y., Li Y., Wang X. (2009). Molecular properties, functions, and potential applications of NAD kinases. Acta Biochim. Biophys. Sin..

[22] Okazaki S., Suzuki A., Mizushima T., Kawano T., Komeda H., Asano Y., Yamane T. (2009). The novel structure of a pyridoxal 5′-phosphate-dependent fold-type I racemase, alpha-amino-epsilon-caprolactam racemase from *Achromobacter obae*. Biochemistry.

[23] Fitzpatrick T.B., Amrhein N., Kappes B., Macheroux P., Tews I., Raschle T. (2007). Two independent routes of de novo vitamin B6 biosynthesis: Not that different after all. Biochem. J..

[24] Schiroli D., Peracchi A. (2015). A subfamily of PLP-dependent enzymes specialized in handling terminal amines. Biochim. Biophys. Acta.

[25] Bisercić M., Feutrier J.Y., Reeves P.R. (1991). Nucleotide sequences of the gnd genes from nine natural isolates of *Escherichia coli*: Evidence of intragenic recombination as a contributing factor in the evolution of the polymorphic gnd locus. J. Bacteriol..

[26] Moccand C., Kaufmann M., Fitzpatrick T.B. (2011). It takes two to tango: Defining an essential second active site in pyridoxal 5′-phosphate synthase. PLoS ONE.

[27] Kim J.H., Kim J., Kim H.J., Sathiyanarayanan G., Bhatia S.K., Song H.S., Choi Y.K., Kim Y.G., Park K., Yang Y.H. (2017). Biotransformation of pyridoxal 5′-phosphate from pyridoxal by pyridoxal kinase (pdxY) to support cadaverine production in *Escherichia coli*. Enzym. Microb. Technol..

[28] Han H., Xu B., Zeng W., Zhou J. (2020). Regulating the biosynthesis of pyridoxal 5′-phosphate with riboswitch to enhance L-DOPA production by *Escherichia coli* whole-cell biotransformation. J. Biotechnol..

[29] Ma W., Cao W., Zhang B., Chen K., Liu Q., Li Y., Ouyang P. (2015). Engineering a pyridoxal 5′-phosphate supply for cadaverine production by using *Escherichia coli* whole-cell biocatalysis. Sci. Rep..

[30] Momany C., Ernst S., Ghosh R., Chang N.L., Hackert M.L. (1995). Crystallographic structure of a PLP-dependent ornithine decarboxylase from Lactobacillus 30a to 3.0 A resolution. J. Mol. Biol..

[31] Oliveira E.F., Cerqueira N.M., Fernandes P.A., Ramos M.J. (2011). Mechanism of formation of the internal aldimine in pyridoxal 5′-phosphate-dependent enzymes. J. Am. Chem. Soc..

[32] Seol E., Sekar B.S., Raj S.M., Park S. (2016). Co-production of hydrogen and ethanol from glucose by modification of glycolytic pathways in *Escherichia coli*—from Embden-Meyerhof-Parnas pathway to pentose phosphate pathway. Biotechnol. J..

[33] Moritz B., Striegel K., De Graaf A.A., Sahm H. (2000). Kinetic properties of the glucose-6-phosphate and 6-phosphogluconate dehydrogenases from *Corynebacterium glutamicum* and their application for predicting pentose phosphate pathway flux in vivo. Eur. J. Biochem..

[34] Hao N., Mu J., Hu N., Xu S., Shen P., Yan M., Li Y., Xu L. (2016). Implication of ornithine acetyltransferase activity on l-ornithine production in *Corynebacterium glutamicum*. Biotechnol. Appl. Biochem..

[35] Hao N., Mu J., Hu N., Xu S., Yan M., Li Y., Guo K., Xu L. (2015). Improvement of L-citrulline production in *Corynebacterium glutamicum* by ornithine acetyltransferase. J. Ind. Microbiol. Biotechnol..

[36] Caldovic L., Tuchman M. (2003). N-acetylglutamate and its changing role through evolution. Biochem. J..

[37] Zhang B., Gao G., Chu X.H., Ye B.C. (2019). Metabolic engineering of *Corynebacterium glutamicum* S9114 to enhance the production of l-ornithine driven by glucose and xylose. Bioresour. Technol..

[38] Kim S.M., Choi B.Y., Ryu Y.S., Jung S.H., Park J.M., Kim G.H., Lee S.K. (2015). Simultaneous utilization of glucose and xylose via novel mechanisms in engineered *Escherichia coli*. Metab. Eng..

[39] Shams Yazdani S., Gonzalez R. (2008). Engineering *Escherichia coli* for the efficient conversion of glycerol to ethanol and co-products. Metab. Eng..

[40] Hawi A.A., Yip H., Sullivan T.S., Digenis G.A. (1988). Development of an HPLC assay for the analysis of tetrafluoroputrescine—A putrescine analog. Anal. Biochem..

