# Supplementary material for: Increasing 1,4-Diaminobutane Production in Escherichia coli by Optimization of Cofactor PLP and NADPH Synthesis
Source: Molecules. 2024 Jun 28;29(13):3094. doi: 10.3390/molecules29133094 (PMC11243127; doi:10.3390/molecules29133094)
Supplement: Supplementary file 1 [file molecules-29-03094-s001.zip › molecules-3034506-supplementary.pdf]

Supplementary material

Table S1 Primers used in this study.

Primer name	Primer sequence
pntAB-NdeI-F	GGAATTCCATATGCGAATTGGCATACCAAGA
pntAB-SpeI-R	GGACTAGTTTACAGAGCTTTCAGGATTGCATCC
ppnk-NdeI-F	GGAATTCCATATGACTGCACCCACGAAC
ppnK-speI-R	GGACTAGTTTACCCCGCTGACCTGGG
dxs-NdeI-F	GGAATTCCATATGAGTTTTGATATTGCCAAATACCCGACCC
dxs-SpeI-R	GGACTAGTTTATGCCAGCCAGGCCTTGATTT
pdxJ-NdeI-F	GGGAATTCCATATGGCTGAATTACTGTTAGGCG
pdxJ-SpeI-R	GGACTAGTTTAGCCACGCGCTTCCAGCATCAGA
zwf-NdeI-F	GGAATTCCATATGGCGGTAACGCAAACAGCCCA
zwf-XhoI-R	CCGCTCGAGTTACTCAAACTCATTCCAGGAACGACC
gnd-NdeI-F	GGAATTCCATATGTCCAAGCAACAGATCGGCG
gnd-XhoI-R	CCGCTCGAGTTAATCCAGCCATTCGGTATGGAACACA
pgl-NdeI-F	GGAATTCCATATGAAGCAAACAGTTTATATCGCCAG
pgl-XhoI-R	CCGCTCGAGTTAGTGTGCGTTAACCACCACCC
tktA-NdeI-F	GGAATTCCATATGTCCTCACGTAAAGAGCTTGCC
tktA-XhoI-R	CCGCTCGAGTTACAGCAGTTCTTTTGCTTTCGC
talB-NdeI-F	GGAATTCCATATGACGGACAAATTGACCTCCCT
talB-XhoI-R	CCGCTCGAGTTACAGCAGATCGCCGATCATTTTTTCC
glpFK-XhoI-R	CCGCTCGAGTTATTCGTCGTGTTCTTCCCACG
glpFK-NdeI-F	GGAATTCCATATGAGTCAAACATCAACCTTGAAAGGCC
tpiA-NdeI-F	GGAATTCCATATGCGACATCCTTTAGTGATGGGT
gapB-B.S-NdeI-F	GGAATTCCATATGAAGGTAAAAGTAGCGATCAACGG
gapB-B.S-XhoI-R	CCGCTCGAGTTATACAGCAGACGGATGTTTCATTCG
iolT1-XhoI-F	CCGCTCGAGTTAGTGCACCTTTCCTTTTCGGATG
iolT1-NdeI-F	GGAATTCCATATGGCTAGTACCTTCATTCAGGCCG
ppgk-XhoI-R	CCGCTCGAGTTATGGGGTGAGGTGTTGGTTTAC
ppgk-NdeI-F	GGAATTCCATATGACTGAGACTGGATTTGGAATTGAT
ODC-F	TATTTCTTCAGTTCAGCCAGTGATGCCGGCCACGATGCGTC

ODC-R	CCCCTATAGTGAGTCGTATTATCAGCTTGTCGACTCCTCCTTTC
argJ-F	GAAAGGAGGAGTCGACAAGCTGATAATACGACTCACTATAGGGG
argJ-R	CAACAATGGCTCGTCACCGTAGGGGTTCCGCGCACATTTC
T7-F	TAGTCTAAACGGGTCTTGAGGGGT
T7-R	AAACACCCCTCAAGACCCGTTTAG

Table S2 List of plasmids

Plasmid	Host strain	Source
pETM6	Gene expression vector,Amp	Peng Xu et al.(2012)
pRSM3	Gene expression vector,Kan	Peng Xu et al.(2012)
pACM4	Gene expression vector,Str	Peng Xu et al.(2012)
pCDM4	Gene expression vector,Cl	Peng Xu et al.(2012)
pTrc99A	Gene expression vector,Amp	Kept in our lab
pETM6-ppnk	pETM6 carrying *ppnK* from *C.glutamicum* 13032,Amp	This study
pETM6-pntAB	pETM6 carrying *pntAB* from *Escherichia coli str. K12 substr. MG1655,*Amp	This study
pETM6-ppnk-pntAB	pETM6 carrying *ppnk and pntAB* Amp	This study
pETM6-zwf	pETM6 carrying *zwf* from *Escherichia coli str. K12 substr. MG1655,*Amp	This study
pETM6-gnd	pETM6 carrying *gnd* from *Escherichia coli str. K12 substr. MG1655,* Amp	This study
pETM6-pgl	pETM6 carrying *pgl* from *Escherichia coli str. K12 substr. MG1655,* Amp	This study
pETM6-zwf-pgl-gnd	pETM6 carrying *zwf*， *pgl* and *gnd,* Amp	This study
pETM6-pdxJ	pETM6 carrying *pdxJ* from *Escherichia coli str. K12 substr. MG1655,*Amp	This study
pETM6-dxs	pETM6 carrying *dxs* from *Escherichia coli str. K12 substr. MG1655*Amp	This study
pETM6-tktA	pETM6 carrying *tktA* from *Escherichia coli str. K12 substr. MG1655,* Amp	This study
pETM6-talB	pETM6 carrying *talB* from *Escherichia coli str. K12 substr. MG1655,* Amp	This study
pETM6-pdxJ-dxs-tktA-talB	*pETM6* carrying *pdxJ, dxs,tktA and talB* Amp	This study
pRSM3-argJ-speC	pRSM3carrying *argJ and speC* Kan	This study

pETM6-zwf-pgl	pETM6 carrying *zwf and pgl*, Amp	This study
pETM6-tktA-talB	pETM6 carrying *tktA and talB* , Amp	This study
pCDM4-pntAB-ppnk	pCDM4 carrying *ppnk* and *pntAB*, Cl	This study
pACM4-zwf-pgl-gnd	pACM4 carrying *zwf*，*pgl* and *gnd*, Str	This study
pETM6-glpFK-tpiA-gapB	pETM6 carrying *glpFK* and *tpiA* from *Escherichia coli str. K12 substr. MG1655*,*gapB* from *Bacillus subtilis subsp. subtilis str. 168*, Amp	This study
pETM6-iolt-ppgk	pETM6 carrying *iolt* and *ppgk* from *Escherichia coli str. K12 substr. MG1655*,Amp	This study
pETM6-xylAB	pETM6 carrying *xylAB* from *Escherichia coli str. K12 substr. MG1655*, Amp	This study
pCDM4-pdxJ-dxs-tktA-talB-pntAB-ppnk	pCDM4 carrying *ppnk ,pntAB ,pdxJ, dxs, tktA and talB Cl*	This study
pTrc99A-speC-argJ	pTrc99A carrying *speC* and *argJ*,Amp	This study
pRSM3-iolT-ppgk	pRSM3 carrying *iolt* and *ppgk*,Amp	This study

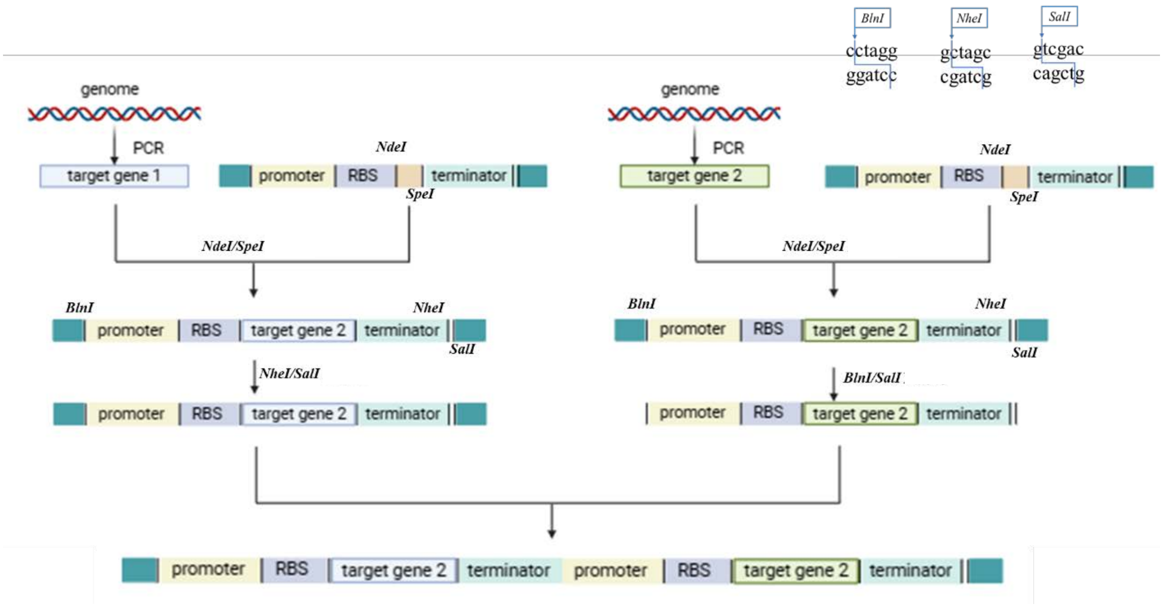

Figure S1 Flow chart of tandem carrier constructed by isocaudamers

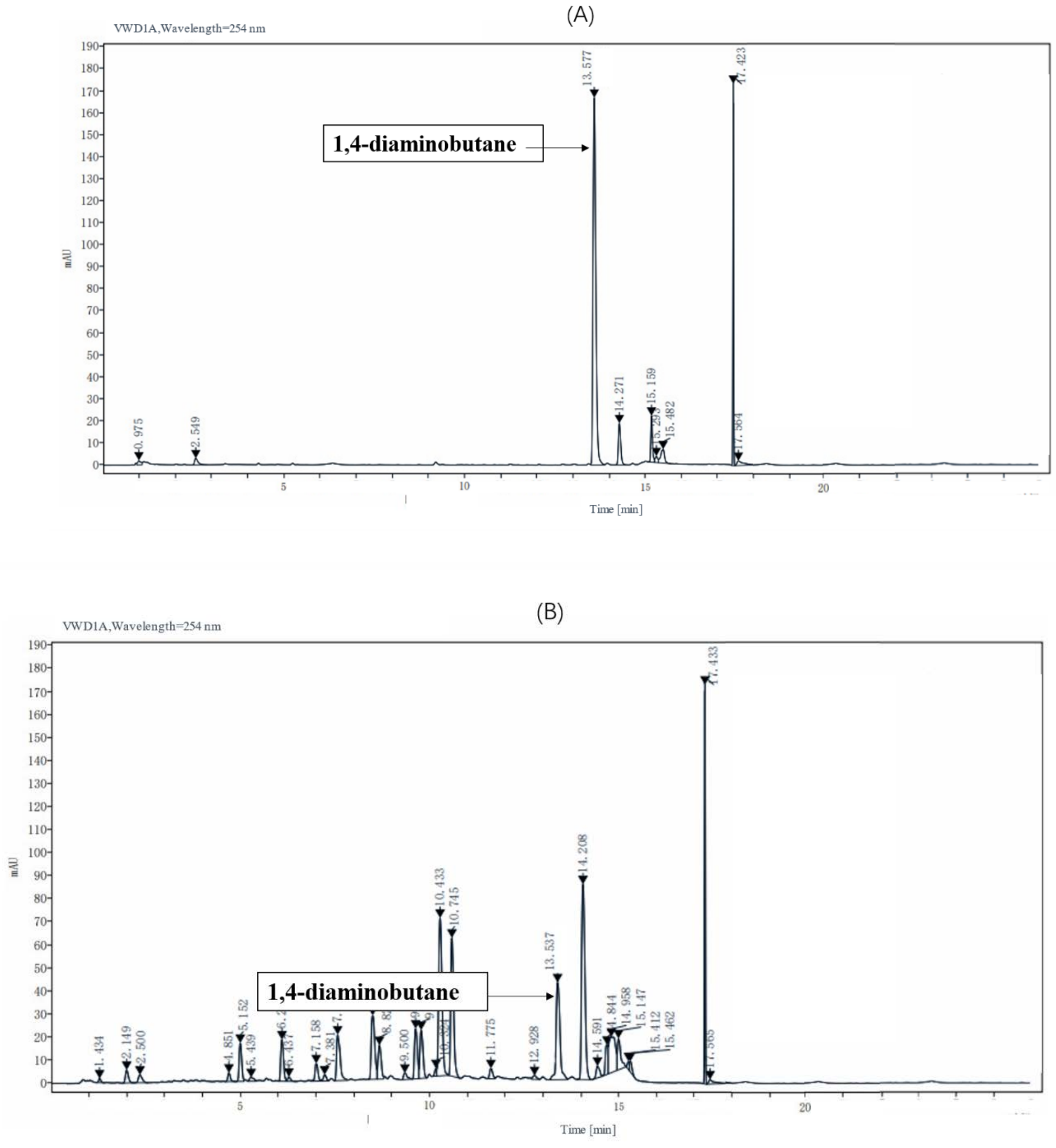

Figure S2 Determination of 1,4-diaminobutane by HPLC. (A) the chromatographic peak of 1,4-diamino-butane standard, which peaked in about 14 min. (B) the chromatographic peak of the sample, which peaked in about 14 min.
